# USEQ: A Short Questionnaire for Satisfaction Evaluation of Virtual Rehabilitation Systems

**DOI:** 10.3390/s17071589

**Published:** 2017-07-07

**Authors:** José-Antonio Gil-Gómez, Pilar Manzano-Hernández, Sergio Albiol-Pérez, Carmen Aula-Valero, Hermenegildo Gil-Gómez, José-Antonio Lozano-Quilis

**Affiliations:** 1 Instituto Universitario de Automática e Informática Industrial, Universitat Politècnica de València, Camino de Vera s/n, 46022 Valencia, Spain; hgil@ai2.upv.es (H.G.-G.); jlozano@upv.es (J.-A.L.-Q.); 2 Hospital S. José, Av. Zaragoza 16, 44001 Teruel, Spain; pilarmanzanoh@hotmail.com (P.M.-H.); aula856@gmail.com (C.A.-V.); 3 Aragón Health Research Institute (IIS Aragón), Universidad de Zaragoza, Ciudad Escolar, 44003 Teruel, Spain; salbiol@unizar.es

**Keywords:** virtual rehabilitation, usability, satisfaction, questionnaire, factor analysis, analysis of principal components

## Abstract

New emerging technologies have proven their efficacy in aiding people in their rehabilitation. The tests that are usually used to evaluate usability (in general) or user satisfaction (in particular) of this technology are not specifically focused on virtual rehabilitation and patients. The objective of this contribution is to present and evaluate the USEQ (User Satisfaction Evaluation Questionnaire). The USEQ is a questionnaire that is designed to properly evaluate the satisfaction of the user (which constitutes part of usability) in virtual rehabilitation systems. Forty patients with balance disorders completed the USEQ after their first session with ABAR (Active Balance Rehabilitation), which is a virtual rehabilitation system that is designed for the rehabilitation of balance disorders. Internal consistency analysis and exploratory factor analysis were carried out to identify the factor structure of the USEQ. The six items of USEQ were significantly associated with each other, and the Cronbach alpha coefficient for the questionnaire was 0.716. In an analysis of the principal components, a one-factor solution was considered to be appropriate. The findings of the study suggest that the USEQ is a reliable questionnaire with adequate internal consistency. With regard to patient perception, the patients found the USEQ to be an easy-to-understand questionnaire with a convenient number of questions.

## 1. Introduction

### 1.1. Usability

Usability is an important quality attribute of a user’s experience when interacting with a system or tool, and it is also an important attribute in helping users to achieve the suggested goals [1]. With regard to HCI (Human–Computer Interface) and usability, Bevan states in [2] that standards related to usability can be categorized as being primarily concerned with the use of the product (effectiveness, efficiency, and satisfaction in a specific context of use).

The categorization of Bevan is coherent with the ISO 9241-11 standard [3,4,5], which describes a widely accepted definition of usability. This standard indicates the rules that are needed in terms of ergonomics, hardware, software, and environments in order to obtain good usability for a product or system. Section 8.1 describes the term usability as “the extent to which a product can be used by specified users to achieve specified goals with effectiveness, efficiency and satisfaction in a specified context of use”.

### 1.2. Usability in Virtual Rehabilitation

One of the promising and emerging fields within rehabilitation therapies for different pathologies is virtual rehabilitation (VRh) [6,7,8,9]. VRh systems are designed to assist clinical specialists and patients in the rehabilitation process [10]. The use of ground-breaking technologies together with the emergence of entertaining and playful virtual environments (VE) have demonstrated promising results in the rehabilitation process [11,12,13,14], improving the adherence to treatments [12]. However, these systems should be tested regarding important aspects such as usability.

Currently, there are different questionnaires that are designed to evaluate usability in general-purpose systems. The best-known usability questionnaire is the system usability scale (SUS) [15,16], which measures the feeling of usability of the users when using computer systems. It is composed of 10 questions with a five-point Likert attitude scale (from strongly disagree to strongly agree). This questionnaire has been used in different domains such as: security software [17], mobile phones [18,19], PDA [20], Social Network sites [21,22], wiki sites [23], serious games [24], or robotics [25]. Even though the SUS questionnaire is not specifically designed for VRh systems, it has also been used for rehabilitation purposes due to the lack of questionnaires that focus on VRh systems. Meldrum et al. [26] tested balance in patients with vestibular and other neurological diseases using VRh and quantified the usability of the Nintendo Wii Fit Plus^®^. Duvinage et al. [27] assessed the usability of a P300 system (using Brain–Computer interfaces) for lower-limb rehabilitation purposes. One considerable advantage of the SUS questionnaire is the reasonable number of questions that are to be answered at the end of the first session. However, the concepts of this questionnaire are too generic (computers, PDAs, Websites, etc.). The main drawback of the SUS questionnaire is that it does not include questions to obtain responses about specific items related to Virtual Rehabilitation.

Another well-known usability questionnaire is VRUSE [28]. Fitzgerald et al. [29] assessed the usability of the E-Yoga system using VRUSE, with the goal of improving postural control and biomechanical alignment of the subjects in a rehabilitation process. The VRUSE evaluates a wide range of concepts: functionality, user input, system output (display), user guidance and help, consistency, flexibility, simulation fidelity, error correction/handling and robustness, sense of immersion/presence, and overall system usability. The main drawback of this test is the large number of questions that the patients are required to answer [28]: the complete questionnaire has 100 questions. This drawback is especially important if the patients involved in a rehabilitation process have neurological and/or cognitive disorders. Other simplified usability questionnaires for VRh with reasonable outcomes are described in [30,31,32,33], but the drawback of these questionnaires is that the internal consistency has not yet been validated.

Kizony et al. [34] published the Short Feedback Questionnaire (SFQ), which is a questionnaire that is related to Witmer and Singer’s Presence Questionnaire [35]. It is composed of eight questions with a five-point Likert attitude scale, and it has been used in virtual reality environments [36,37,38]. The SFQ questionnaire evaluates the user’s sense of presence, perceived difficulty of the task, and any discomfort that users may have felt during the experience. This questionnaire does not focus on VRh systems.

To our knowledge, there are no validated questionnaires for testing usability or satisfaction of virtual rehabilitation systems. A questionnaire for this purpose must have a reasonable number of questions and internal consistency reliability.

Following the definitions of usability in [2,3,4,5], usability can be divided into three components: efficiency, effectiveness, and satisfaction. Focusing on VRh, efficiency and effectiveness can usually be measured through a clinical trial. With a classical clinical trial, we can compare an experimental group (using a VRh system) with a control group (following a traditional rehabilitation program) by evaluating efficacy and comparing the recovery level of the two groups. With regard to effectiveness, we can measure, for instance, the number of sessions that each group needs to reach a certain level. However, the third component of usability, satisfaction, cannot be evaluated in the same way as efficiency and effectiveness: a reliable and consistent questionnaire (with an adequate number of questions) is necessary to measure the satisfaction of the users.

The aim of the present study is to introduce the USEQ, a user satisfaction questionnaire that is specifically designed to evaluate satisfaction with virtual rehabilitation systems, and to validate their reliability by analyzing their internal consistency.

## 2. USEQ: The User Satisfaction Evaluation Questionnaire

### 2.1. SEQ: The Suitability Evaluation Questionnaire

In [39], the SEQ was introduced as a 14-question questionnaire that is designed to test items such as satisfaction, acceptance, and security of use in virtual rehabilitation systems. The SEQ was designed by a multidisciplinary team of clinical and technical experts. Factors such as the length of the questionnaire, the type of questions to be asked and what to ask were taken into account in the design of the questionnaire. For the length of the questionnaire, the clinical experts that collaborated in the design of the SEQ estimated that a maximum of 15 questions would be an acceptable length for patients.

For the type of questions, the designers of the SEQ considered 13 questions with a five-point Likert Scale, plus an open-ended question offering patients the possibility to add comments if necessary. The SEQ has a five-point Likert Scale questions (instead of other options such as seven-point Likert Scale questions) because the authors considered five options of answers to be good enough, and, also, it is coherent with the main usability questionnaires that are currently being used: SUS [15], VRUSE [28], and SFQ [34] also use five-point Likert Scale questions.

For what to ask about, the designers of the SEQ composed the questions taking into account the usability questionnaires available and their own experience, both in the technical and in the clinical field.

A previous study evaluating the suitability of virtual rehabilitation for the elderly was carried out using the SEQ [40]. The SEQ was used to evaluate the ABAR (Active Balance Rehabilitation) system, the VRh system that is used in this study. The study presented in [40] allowed the evaluation of the perceived length and difficulty of the SEQ. In [40], the patients completed the questionnaire without any problems. None of the patients considered the questionnaire to be too long. The main drawback of SEQ is that it is composed of different dimensions; therefore, it is not possible to evaluate their internal consistency.

### 2.2. USEQ: Questions

The USEQ questionnaire is composed of the set of questions in the SEQ that evaluate satisfaction. The USEQ has six questions with a five-point Likert Scale. The questions and their scores are shown in Table 1.

The total score of the USEQ questionnaire ranges from 6 (poor satisfaction) to 30 (excellent satisfaction). To calculate this total score, we consider all of the questions to be positive, except for Q5, which is considered to be a negative question. The numerical value of the positive questions is used to calculate the score (for instance, if the patient selects 4 in Q1, then 4 is added to the total score). The negative question subtracts the numerical value of the response from 6 and then adds this result to the total score (for instance, if the patient selects 2 in Q5, then 4 is added to the total score).

## 3. Study Design

### 3.1. Subjects

Patients who had balance disorders and were attending a VRh program were the potential participants in this study. The diagnoses of these patients include stroke, multiple sclerosis, meningioma, subdural hematoma, cervical myelopathy, Guillain-Barré syndrome, Parkinson disease, brain tumors, and vestibular pathology. 

The inclusion criteria were: (1) signed written informed consent; (2) age >17; (3) balance disorders evidenced by clinical balance scales; (4) normal cognitive functions; (5) ability to follow instructions (mini-mental state examination [41] >24). The exclusion criteria were: (1) dementia; (2) hemispatial neglect; (3) visual deficit; (4) severe hearing impairment; (5) unsolved acute trauma injury.

### 3.2. Study Interventions

**Figure 1:**
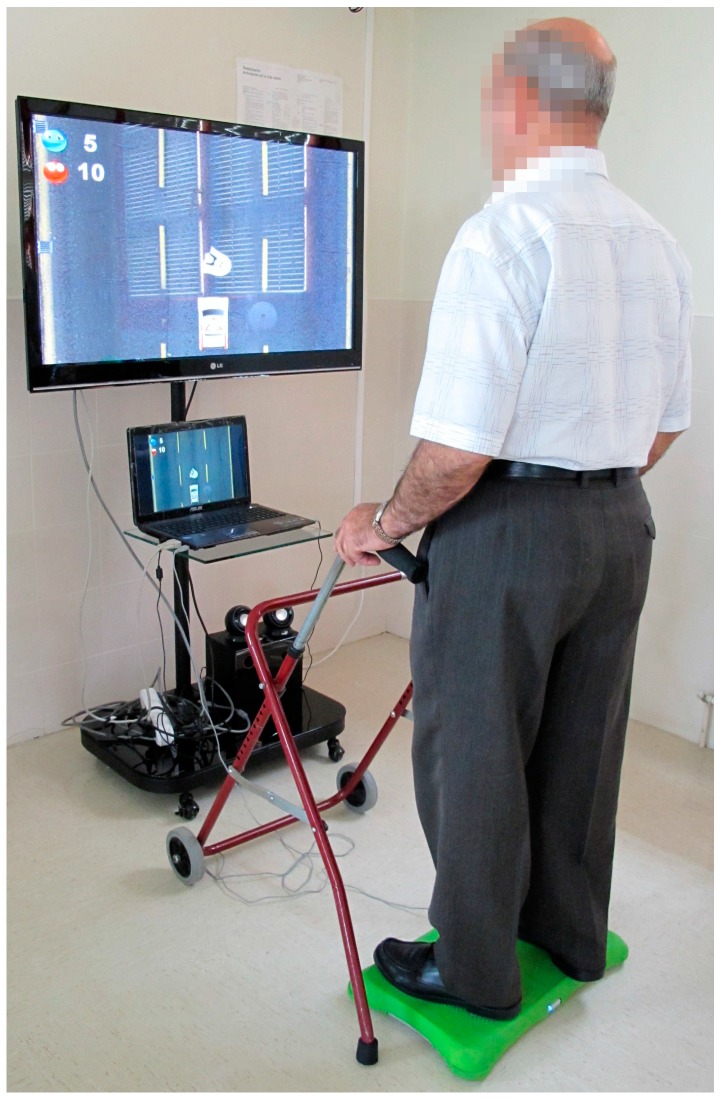
Patient interacting with the ABAR system.

In the study, the USEQ is used to test satisfaction of the ABAR system [42]. The ABAR system is a VRh system that is specifically designed to recover balance (Figure 1).

ABAR integrates the Nintendo^®^ Wii Balance Board (WBB) (Nintendo, Kyoto, Japan) for the interaction of the patient. WBB is a low-cost, widely-available device that allows the center-of-pressure of the patient to be obtained.

Five different games can be selected in ABAR to recover balance, in both sitting and the standing positions.

### 3.3. Study Procedures

The study was conducted in a specialized rehabilitation facility of a hospital under clinical supervision. Each patient completed the USEQ after the first session with ABAR. Each session with the system lasted 30 min; each session mixed periods of playing and resting, according to the specialist’s indications. A member of our team was with the patients while they were answering the questionnaire. 

### 3.4. Outcome Measures

The primary outcome measures were provided by the questionnaire. The scores for the questions of the USEQ allowed us to carry out the statistical analysis as described in Section 4.

Secondary measures were obtained when the patients completed the USEQ. At this stage, we had informal conversations about the USEQ with patients after the completion of the questionnaire. Although the informal conversations with patients are a subjective source of information, they provided us with responses to questions that are related to perceived questionnaire length or perceived questionnaire difficulty.

### 3.5. Data Analysis

Data analysis was carried out with SPSS for Windows, version 15 (SPSS Inc., Chicago, IL, USA) on a standard PC. To test the internal consistency reliability, we used Cronbach’s alpha [43]. Cronbach’s alpha is a coefficient of internal consistency that is commonly used to estimate the reliability of a test.

For sampling adequacy, the Kaiser-Meyer-Olkin (KMO) index and Bartlett’s test of sphericity were calculated. While the KMO index ranges from 0 to 1, adequate sample size is accepted for a value over 0.5. For factor analysis to be considered suitable, Bartlett’s test of sphericity must be less than 0.05.

To identify the factor structure of the USEQ, we carried out an exploratory factor analysis (analysis of principal components), retaining components with eigenvalues greater than 1; in addition, we carried out a scree plot inspection. For the correlations between the items and the factor, we used unrotated factor loadings above 0.3. 

## 4. Results

### 4.1. Sample Characteristics

In the study period, 198 patients who had balance problems and were attending a rehabilitation program in the clinical facilities were the potential participants in this study. Of these, 108 patients had unsolved acute trauma injuries, 36 patients had cognitive impairment, 5 patients had visual deficit or severe hearing impairment, and 9 patients refused to participate in the study. A final sample of 40 patients fulfilled the inclusion–exclusion criteria, and were included in the study.

Table 2 shows information summarizing the characteristics of the patients.

In the final sample, 19 patients were male and 21 patients were female. Most of the patients were elderly: 80% of the patients were older than 65 years old and the average age was 74.35 (SD 14.59) years old. Based on the post-injury time [44], 10 patients were post-acute (0–5 months post-injury), 11 patients were acute (6–23 months post-injury), and 19 patients were chronic (24 months or more post-injury). Most of the patients came from an urban background (75%), and 25% came from a rural background. With regard to the level of studies, 85% of the patients had completed primary (60%) or secondary (25%) studies whereas only three patients had completed higher studies and only three patients had not completed any studies.

### 4.2. USEQ Scores

The results corresponding to the USEQ question evaluation are presented in Table 3.

The mean USEQ score was 25.80 (SD 3.589). The scale mean if the item is deleted was measured for all the items, ranging from 21.10 to 22.00. 

### 4.3. Item-Total Correlation and Cronbach’s Alpha

To carry out the item analysis for selecting items for inclusion in the scale, we used the corrected item-total correlation. In this way, we avoid the problem of performing the correlation of an item with the total of the scale, considering that this total includes the value of the item whereby that correlation would be skewed. The corrected item-total correlation values ranged from 0.321 to 0.666 (Table 3).

The Cronbach’s alpha value for the complete scale was 0.716. Cronbach’s alpha if the item is deleted was calculated for all six items, and none of the values were above Cronbach’s alpha for complete scale (Table 3).

### 4.4. Factor Structure

The KMO index of sampling adequacy was 0.60. Bartlett’s test of sphericity was significant (*p* < 0.001). The analysis of principal components indicated two components with an eigenvalue greater than 1, which accounted for 65.777% of the total variance. The first of the components had all six items (Table 4), and it explains 42.869% of the variance; the items of the first component had a correlation with the factor that ranged from 0.468 to 0.816. The second component had only four items, only two of which had factor loadings greater than 0.5 (Table 4).

**Figure 2:**
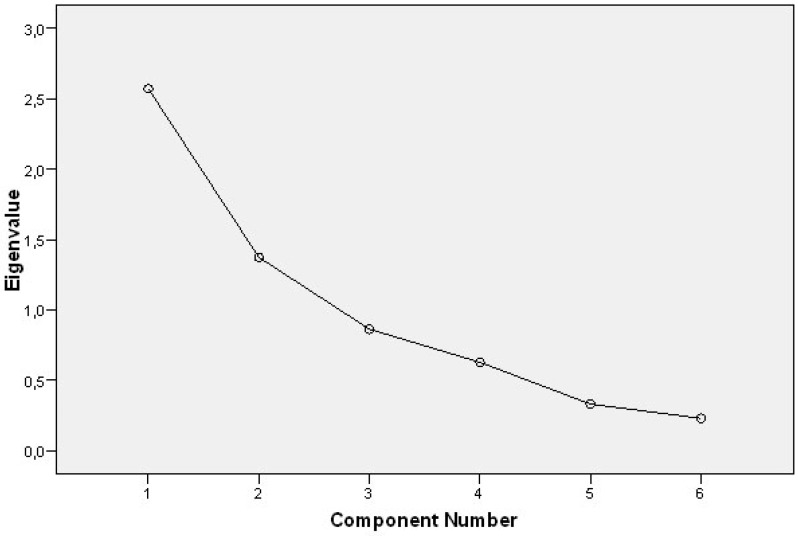
Scree plot showing eigenvalues of components after analysis of the principal components. Only two components had an eigenvalue greater than 1. The scree plot did not present a clear point of inflexion, although a closer inspection indicated a slight inflexion in the second component.

The scree plot (Figure 2) did not reveal a clear point of inflexion, but the sharpest angle is placed in the second component.

### 4.5. Informal Conversations

The informal interview after the completion of the questionnaire is a subjective source of data, but it is always very interesting to know the opinions of patients. The patients considered that the questionnaire was short and they were not distracted when answering the questions. The patients also perceived the USEQ to be an easy-to-understand questionnaire.

## 5. Discussion and Conclusions

Despite the fact that virtual rehabilitation is an emerging field that shows great potential, with many studies in recent years, there are no specific usability or satisfaction questionnaires with validated internal consistency for virtual rehabilitation systems. The USEQ is a questionnaire that is designed to evaluate satisfaction, which is part of usability, in virtual rehabilitation systems. This study has addressed the factor structure and internal consistency of the USEQ. Analysis of item-total correlation (Table 3) suggested that all items correlated well with the overall scale because all of the values were above 0.3. Therefore, the results suggest that the USEQ is a reliable questionnaire.

With regard to the Cronbach’s alpha evaluation, Cronbach explains in [43] how this coefficient should be interpreted. Cronbach indicates that alpha values greater than or equal to 0.7, in general indicate good internal consistency. On the other hand, if Cronbach’s alpha is too high, it may suggest redundancies because some items are testing the same question but in a different way. Streiner [45] recommends a maximum alpha value of 0.9.

In general, although no accurate ranges exist to classify the Cronbach alpha coefficient, an alpha coefficient ranging between 0.7 and 0.9 is considered to be acceptable. The Cronbach alpha coefficient for the USEQ was 0.716; therefore, this indicates adequate internal consistency.

The increase in Cronbach’s alpha when an item is deleted indicates that the item could probably be removed from the scale. For the USEQ, the results of the study showed that the Cronbach alpha values if the item is deleted were minor for all six items (Table 3). Therefore, we kept all of the questions of the USEQ.

Regardless of the fact that an analysis of the principal components showed two factors with an eigenvalue greater than 1, the features of the second factor (Table 4) and the scree plot inspection (Figure 2) suggest that only the first factor (which includes all six questions) can be considered to be appropriate. Therefore, a one-factor solution was considered to be appropriate, which accounted for 42.869% of the total variance. As shown in Table 4, all items had a correlation greater than 0.4 with the factor, which implies that they are probably meaningful. Thus, the factor under consideration represents ‘user satisfaction’ because all of the items were designed to measure user satisfaction with the system, and it has a Cronbach alpha coefficient of 0.716.

Other similar studies that evaluate the internal consistency of tests with good results show comparable values. 

In [46], the authors evaluated HARUS (Handheld Augmented Reality Usability Scale), a questionnaire composed of 16 statements where users rate their agreement by using a seven-point Likert scale. HARUS has a two-factor structure. Statements 1 to 8 are measures of manipulability, while statements 9 to 16 are measures of comprehensibility. The authors confirm that the separate manipulability and comprehensibility scales have good internal consistency because they obtained alpha values between 0.71 and 0.83 in all their experiments. 

Fackrell et al. [47] evaluated the validity and reliability of the Hyperacusis Questionnaire. The authors also specify α > 0.7 as reliability criteria for the scales evaluated.

Although the Cronbach alpha coefficient in this study is acceptable (0.716), it is just above the lower value of the range of values considered to be adequate (0.7 ≤ α ≤ 0.9). Several factors related to the sample may have influenced this result. We hypothesize that the size of the sample and/or the age of the patients (mostly elderly) could have influenced the result. As we suggest later, further studies with different samples can allow us to check how these factors influence the results.

In the study, the patients observed that the USEQ has a convenient number of questions. They also considered the questions in the USEQ to be clear. This perception of the questionnaire by the patients is especially interesting considering the age of the patients enrolled in the study (most were elderly) and their level of studies (67.50% had only primary studies or less). With younger patients, and/or with patients with a higher level of studies, it is expected that the perception of the questionnaire will be even more favorable.

With regard to the results for the evaluated system (ABAR), the USEQ score was 25.80. Since the scale ranges from 6 (lower satisfaction) to 30 (higher user satisfaction), the score suggests that the satisfaction perceived by patients was very high. With regard to the characteristics of the sample, it is necessary to pay attention to some points. The sample includes a heterogeneous population from the point of view of their disabilities. This is due to the VRh system used for the evaluation: ABAR is a VRh system designed to help in the recovery of balance, and there are many different disabilities that cause balance disorders. Moreover, these disabilities are most common among the elderly and, consequently, the patients of the sample tend to be seniors. Future studies that evaluate the USEQ with a younger population and/or with a homogeneous population with respect to their disabilities would be interesting, in order to compare their results to the results of this study.

With regard to the sample size, the main drawback of the study is that it is a bit limited to perform factor analytic studies. However, but it was impossible to add more patients that fulfilled the inclusion–exclusion criteria. In the future, further studies with larger samples will allow the results of this study to be compared.

Based on the results obtained, we do not suggest any changes in the items of the USEQ. From an objective point of view, this is for especially two reasons. First, the results confirmed a one-factor solution and adequate internal consistency. Second, the results support that all the items are necessary: first, because the results of the study showed that the Cronbach alpha values, if an item was deleted, were minor for all six items, and, second, because the Cronbach alpha coefficient is below 0.9 (a high value of alpha may suggest redundancies and indicate that the length of the questionnaire should be shortened). From a subjective point of view, the positive perception of the USEQ by the patients support our suggestion.

In summary, the USEQ presented in this study is an easy-to-understand questionnaire that has an appropriate number of questions that are correlated with each other. The USEQ is a reliable and useful tool for properly evaluating the satisfaction of the user (which is part of usability).

## Figures and Tables

**Table 1:** The USEQ (User Satisfaction Evaluation Questionnaire).

Question	Response
	Not at All–Very Much
Q1. Did you enjoy your experience with the system?	1 2 3 4 5
Q2. Were you successful using the system?	1 2 3 4 5
Q3. Were you able to control the system?	1 2 3 4 5
Q4. Is the information provided by the system clear?	1 2 3 4 5
Q5. Did you feel discomfort during your experience with the system?	1 2 3 4 5
Q6. Do you think that this system will be helpful for your rehabilitation?	1 2 3 4 5

**Table 2:** Characteristics of the participants.

	Mean	SD
Age (years)	74.25	14.59
Duration of the balance disorders (years)	3.39	5.51
	**n**	**%**
Sex		
Male	19	47.50
Female	21	52.50
Chronicity		
Postacute (0–5 months post-injury)	10	25.00
Acute (6–23 months post-injury)	11	27.50
Chronic (24 months or more post-injury)	19	47.50
Background domicile		
Urban	30	75.00
Rural	10	25.00
Level of studies		
No studies	3	7.50
Primary studies	24	60.00
Secondary studies	10	25.00
Higher studies	3	7.50

**Table 3:** Score on questions of the USEQ. Corrected Item-Total Correlation. Cronbach’s Alpha if item is deleted.

Question	Mean	SD	Scale Mean if Item Deleted	Corrected Item-Total Correlation	Cronbach’s Alpha if Item Deleted
Q1	4.43	0.874	21.38	0.326	0.712
Q2	4.23	1.097	21.58	0.666	0.597
Q3	3.80	1.224	22.00	0.572	0.637
Q4	4.65	0.662	21.15	0.404	0.694
6-Q5	4.70	0.608	21.10	0.496	0.678
Q6	4.00	0.961	21.80	0.321	0.716

**Table 4:** Component matrix. This table contains the correlations between the items and the factors with an eigenvalue greater than 1. The percentage of variance explained by each factor is also shown.

	Component	
	1	2
Questions		
Q2	0.816	
Q3	0.780	
Q5	0.669	0.647
Q4	0.582	−0.414
Q6	0.541	0.672
Q1	0.468	−0.490
Variance explained (%)	42.869%	22.908%

## References

[1] Nielsen J. (2008). Usability Engineering.

[2] Bevan N. (2001). International standards for HCI and usability. Int. J. Hum. Comput. Stud..

[3] Abran A., Khelifi A., Suryn W., Seffah A. (2003). Usability meanings and interpretations in ISO standards. Softw. Qual. J..

[4] ISO 9241–11 Ergonomic Requirements for Office Work with Visual Display Terminals (VDTs) Part 11: Guidance on Usability. http://www.iso.org/iso/catalogue_detail.htm?csnumber=16883.

[5] Jokela T., Iivari N., Matero J., Karukka M. (2003). The standard of user-centered design and the standard definition of usability: analyzing ISO 13407 against ISO 9241-11. Proceedings of the Latin American Conference on Human-Computer Interaction.

[6] Holden M.K. (2005). Virtual environments for motor rehabilitation: Review. Cyberpsychol. Behav..

[7] Sveistrup H. (2004). Motor rehabilitation using virtual reality. J. Neuroeng. Rehabil..

[8] Lange B., Koenig S., Chang C.Y., McConnell E., Suma E., Bolas M., Rizzo A. (2012). Designing informed game-based rehabilitation tasks leveraging advances in virtual reality. Disabil. Rehabil..

[9] Lewis G.N., Rosie J.A. (2012). Virtual reality games for movement rehabilitation in neurological conditions: How do we meet the needs and expectations of the users?. Disabil. Rehabil..

[10] Singh D.K., Mohd Nordin N.A., Abd Aziz N.A., Lim B.K., Soh L.C. (2013). Effects of substituting a portion of standard physiotherapy time with virtual reality games among community-dwelling stroke survivors. BMC Neurol..

[11] Green D., Wilson P.H. (2012). Use of virtual reality in rehabilitation of movement in children with hemiplegia-a multiple case study evaluation. Disabil. Rehabil..

[12] Meldrum D., Herdman S., Moloney R., Murray D., Duffy D., Malone K., French H., Hone S., Conroy R., McConn-Walsh R. (2012). Effectiveness of conventional versus virtual reality based vestibular rehabilitation in the treatment of dizziness, gait and balance impairment in adults with unilateral peripheral vestibular loss: a randomized controlled trial. BMC Ear Nose Throat Disord..

[13] Gil-Gómez J.A., Lloréns R., Alcañiz M., Colomer C. (2011). Effectiveness of a Wii balance board-based system (eBaViR) for balance rehabilitation: A pilot randomized clinical trial in patients with acquired brain injury. J. NeuroEng. Rehabil..

[14] Albiol-Pérez S., Gil-Gómez J.A., Lloréns R., Alcañiz M., Font C.C. (2014). The role of virtual motor rehabilitation: A quantitative analysis between acute and chronic patients with acquired brain injury. IEEE J. Biomed. Health Inform..

[15] Brooke J., Jordan P.W., Thomas B., Weerdmeester B.A., McClelland A.L. (1996). SUS: A “quick and dirty” usability scale. Usability Evaluation in Industry.

[16] Brooke J. (2013). SUS: A Retrospective. J. Usabil. Stud..

[17] DeWitt A.J., Kuljis J. (2006). Aligning usability and security: A usability study of Polaris. Proceedings of the 2nd Symposium on Usable Privacy and Security.

[18] Jokela T., Koivumaa J., Pirkola J., Salminen P., Kantola N. (2006). Methods for quantitative usability requirements: a case study on the development of the user interface of a mobile phone. Pers. Ubiquitous Comput..

[19] Vermeulen J., Neyens J.C., Spreeuwenberg M.D., van Rossum E., Sipers W., Habets H., Hewson D.J., de Witte L.P. (2013). User-centered development and testing of a monitoring system that provides feedback regarding physical functioning to elderly people. Patient Preference Adher..

[20] Költringer T., Grechenig T. (2004). Comparing the immediate usability of graffiti 2 and virtual keyboard. Proceedings of CHI ’04 Extended Abstracts on Human Factors in Computing Systems.

[21] Tsung-han T., Fong-gong W., Yu-Hsiu H. (2013). The Survey of Usability Evaluation in Social Network Sites’ Reply Mechanism. Proceedings of the International Conference on Universal Access in Human-Computer Interaction.

[22] Duffy S.A., Fowler K.E., Flanagan P.S., Ronis D.L., Ewing L.A., Waltje A.H. (2013). The development of the tobacco tactics website. JMIR Res. Protoc..

[23] Mathew D., McKibbon K.A., Lokker C., Colquhoun H. (2014). Engaging With a Wiki Related to Knowledge Translation: A Survey of WhatisKT Wiki Users. J. Med. Internet. Res..

[24] Tolentino G.P., Battaglini C., Pereira A.C.V., de Oliveria R.J., de Paula M.G.M. (2011). Usability of Serious Games for Health. Proceedings of the 3rd International Conference on Games and Virtual Worlds for Serious Applications.

[25] Mattos L.S., Deshpande N., Barresi G., Guastini L., Peretti G. (2013). A novel computerized surgeon-machine interface for robot-assisted laser phonomicrosurgery. Laryngoscope.

[26] Meldrum D., Glennon A., Herdman S., Murray D., McConn-Walsh R. (2012). Virtual reality rehabilitation of balance: assessment of the usability of the Nintendo Wii(^®^) Fit Plus. Disabil. Rehabil. Assist. Technol..

[27] Duvinage M., Castermans T., Petieau M., Seetharaman K., Hoellinger T., Cheron G., Dutoit T. (2012). A subjective assessment of a P300 BCI system for lower-limb rehabilitation purposes. Proceedings of the 34th Annual International Conference of the IEEE Engineering in Medicine and Biology Society.

[28] Kalawsky R.S. (1999). VRUSE–A computerised diagnostic tool: For usability evaluation of virtual/synthetic environment systems. Appl. Ergon..

[29] Fitzgerald D., Kelly D., Ward T., Markham C., Caulfield B. (2008). Usability Evaluation of E-Motion: A Virtual Rehabilitation System Designed to Demonstrate, Instruct and Monitor a Therapeutic Exercise Programme. Proceedings of the Virtual Rehabilitation Conference.

[30] Jeon W., Clamann M., Zhu B., Gil G.H., Kaber D., Rebelo F., Soares M.M. (2012). Usability evaluation of a virtual reality system for motor skill training. Advances in Usability Evaluation Part II.

[31] Cameirão M.S., Badia S.B., Oller E.D., Verschure P.F. (2010). Neurorehabilitation using the virtual reality based Rehabilitation Gaming System: methodology, design, psychometrics, usability and validation. J. Neuroeng. Rehabil..

[32] Regenbrecht H., Hoermann S., McGregor G., Dixon B., Franz E., Ott C., Hale L., Schubert T., Hoermann J. (2012). Visual manipulations for motor rehabilitation. Comput. Graph..

[33] Shin J.H., Ryu H., Jang S.H. (2014). A task-specific interactive game-based virtual reality rehabilitation system for patients with stroke: A usability test and two clinical experiments. J. Neuroeng. Rehabil..

[34] Kizony R., Katz N., Weiss P.L. (2003). Adapting an immersive virtual reality system for rehabilitation. J. Vis. Comput. Animat..

[35] Witmer B.G., Singer M.J. (1998). Measuring Presence in Virtual Environments: A Presence Questionnaire. Presence Teleoper. Virtual Environ..

[36] Kizony R., Katz N., Rand D., Weiss P.L. (2006). Short Feedback Questionnaire (SFQ) to enhance client-centered participation in virtual environments. CyberPsychol. Behav..

[37] Kizony R., Raz L., Katz N., Weingarden H., Weiss P.L. (2005). Using a video projected VR system for patients with spinal cord injury. J. Rehabil. Res. Dev..

[38] Iman B., Miller W.C., McLaren L., Chapman P., Finlayson H. (2013). Feasibility of the Nintendo WiiFit™ for improving walking in individuals with a lower limb amputation. SAGE Open Med..

[39] Gil-Gómez J.A., Manzano-Hernández P., Albiol-Pérez S., Aula-Valero C., Gil-Gómez H., Lozano-Quilis J.A. SEQ: Suitability evaluation questionnaire for virtual rehabilitation systems. Application in a virtual rehabilitation system for balance rehabilitation. Proceedings of the 7th International Conference on Pervasive Computing Technologies for Healthcare.

[40] Muñoz Tomás M.T., Gil-Gómez J.A., Gil-Gómez H., Lozano-Quilis J.A., Albiol-Pérez S., Forcano García M. (2013). Suitability of virtual rehabilitation for the elderly: A study of a virtual rehabilitation system using the SEQ. Eur. Geriatr. Med..

[41] Folstein M.F., Folstein S., Mchugh P.R. (1975). Mini-Mental State: A practical method for grading the cognitive state of patients for the clinicians. J. Psychiatr. Res..

[42] Forcano García M., Albiol-Pérez S., Aula Valero M.C., Gil-Gómez J.A., Solsona Hernández S., Manzano Hernández P. (2013). Balance virtual rehabilitation in the elderly: The use of the “ABAR” system. Eur. Geriatr. Med..

[43] Cronbach L.J. (1951). Coefficient alpha and the internal structure of tests. Psychometrika.

[44] Babikian T., Asarnow R. (2009). Neurocognitive outcomes and recovery after pediatric TBI: Meta-analytic review of the literature. Neuropsychology.

[45] Streiner D.L. (2003). Starting at the beginning: an introduction to coefficient alpha and internal consistency. J. Personal. Assess..

[46] Santos M.E.C., Taketomi T., Sandor C., Polvi J., Yamamoto G., Kato H. (2014). A usability scale for handheld augmented reality. Proceedings of the 20th ACM Symposium on Virtual Reality Software and Technology.

[47] Fackrell K., Fearnley C., Hoare D.J., Sereda M. (2015). Hyperacusis Questionnaire as a Tool for Measuring Hypersensitivity to Sound in a Tinnitus Research Population. BioMed Res. Int..

